# Prospective randomized trial of iliohypogastric-ilioinguinal nerve block on post-operative morphine use after inpatient surgery of the female reproductive tract

**DOI:** 10.1186/1477-5751-7-11

**Published:** 2008-11-28

**Authors:** Salim A Wehbe, Labib M Ghulmiyyah, El-Khawand H Dominique, Sarah L Hosford, Carole M Ehleben, Steven L Saltzman, Eric Scott Sills

**Affiliations:** 1 Department of Obstetrics & Gynecology, Atlanta Medical Center, Atlanta, Georgia, USA; 2 Maternal-Fetal Medicine Division, Department of Obstetrics & Gynecology, American University of Beirut Medical Center; Beirut, Lebanon; 3 Department of Obstetrics & Gynecology, School of Medicine, Louisiana State University Health Sciences Center, New Orleans, Louisiana, USA; 4 The Sims Institute/Sims International Fertility Clinic, Department of Obstetrics & Gynaecology, School of Medicine, Royal College of Surgeons in Ireland; Dublin, Ireland; 5 Department of Obstetrics & Gynecology, Alpert Medical School, Brown University; Providence RI, USA

## Abstract

**Objective:**

To determine the impact of pre-operative and intra-operative ilioinguinal and iliohypogastric nerve block on post-operative analgesic utilization and length of stay (LOS).

**Methods:**

We conducted a prospective randomized double-blind placebo controlled trial to assess effectiveness of ilioinguinal-iliohypogastric nerve block (IINB) on post-operative morphine consumption in female study patients (*n *= 60). Patients undergoing laparotomy via Pfannenstiel incision received injection of either 0.5% bupivacaine + 5 mcg/ml epinephrine for IINB (Group I, *n *= 28) or saline of equivalent volume given to the same site (Group II, *n *= 32). All injections were placed before the skin incision and after closure of rectus fascia via direct infiltration. Measured outcomes were post-operative morphine consumption (and associated side-effects), visual analogue pain scores, and hospital length of stay (LOS).

**Results:**

No difference in morphine use was observed between the two groups (47.3 mg in Group I vs. 45.9 mg in Group II; *p *= 0.85). There was a trend toward lower pain scores after surgery in Group I, but this was not statistically significant. The mean time to initiate oral narcotics was also similar, 23.3 h in Group I and 22.8 h in Group II (*p *= 0.7). LOS was somewhat shorter in Group I compared to Group II, but this difference was not statistically significant (*p *= 0.8). Side-effects occurred with similar frequency in both study groups.

**Conclusion:**

In this population of patients undergoing inpatient surgery of the female reproductive tract, utilization of post-operative narcotics was not significantly influenced by IINB. Pain scores and LOS were also apparently unaffected by IINB, indicating a need for additional properly controlled prospective studies to identify alternative methods to optimize post-surgical pain management and reduce LOS.

## Introduction

In current surgical practice, laparotomy performed through a Pfannensteil incision is one of the most common operations involving the female abdomen [1]; effective post-operative analgesia is essential in such cases. The advent of various multimodal analgesia techniques has greatly facilitated the management of postoperative pain [2,3], and i.v. morphine has emerged as the most widely used and cost-effective agent. Augmentation of i.v. analgesia has been achieved with regional nerve blockade, particularly for patients undergoing hysterectomy [4] or Cesarean delivery [5]. However, the potential role for combined ilioinguinal-iliohypogastric nerve block in the setting of less complicated gynecologic procedures remains unclear.

Since others have studied preincisional and post-operative analgesia with placebo (saline) controls to examine either standard nerve block or direct infiltration of the surgical site [6], we speculated that a multi-stage nerve block (where epinephrine is added to bupivacaine) might offer reduced untoward effects of narcotics, earlier mobilization and shorter post-operative hospitalization. Therefore, our prospective investigation sought to assess combined preincisional and intraoperative/preclosure analgesia with bupivacaine + epinephrine against placebo in a study population of female patients undergoing laparotomy via Pfannensteil incision.

## Methods

### Subjects and randomization

**Figure 1:**
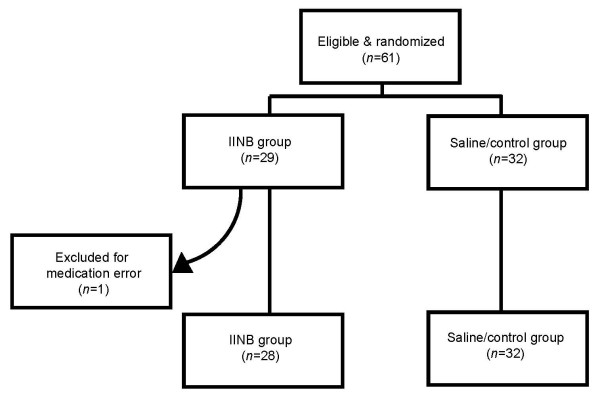
Patient allocation schematic for randomized, placebo-controlled trial of ilioinguinal-iliohypogastric nerve block (IINB).

The investigation enrolled patients during a ten-month period ending May 2005 at Atlanta Medical Center, a large urban teaching affiliate of the Medical College of Georgia, after institutional review board approval. Written informed consent was obtained from all study participants who were randomized as shown in Figure 1. All patients underwent laparotomy via Pfannensteil incision for gynecologic indications summarized in Table 1. Patients were excluded if they reported an allergy to local anesthetics or peptic ulcer disease, renal or liver disease, progressive neurological condition, infection at planned site of the IINB, or history of substance abuse. No patients receiving spinal or epidural anesthesia were enrolled. All patients had standardized preoperative and postoperative orders; no oral or intravenous analgesics were administered preoperatively. Standard general endotracheal anesthesia was performed under supervision of an attending anesthesiologist. Fentanyl was the only analgesic to be used during surgery, with the final dose being given ≥30 min before the end of the procedure.

**Table 1:** Distribution of preoperative indications for surgery among patients randomized either to ilioinguinal-iliohypogastric nerve block (Group I) or saline control (Group II).

	IINB Group I *n *= 28	Saline/controls Group II *n *= 32
Leiomyoma	14 (50)	18 (56.3)
Adenomyosis	2 (7.1)	6 (18.8)
Endometriosis	2 (7.1)	4 (12.5)
Ovarian cyst	1 (3.6)	1 (3.1)
Cervical carcinoma	3 (10.7)	1 (3.1)
Endometrial hyperplasia/carcinoma	2 (7.1)	1 (3.1)
CPP/DUB	5 (17.9)	1 (3.1)

*Note*: Data presented as patient number and (%). CPP/DUB = chronic pelvic pain/dysfunctional uterine bleeding. Totals exceed number enrolled because some patients had multiple pre-operative diagnoses.

Postoperative intravenous patient-controlled analgesia (PCA) was provided for all study patients with basal morphine sulfate rate set at 2 mg. Lockout interval was six minutes, maximum morphine dose was established at 12 mg/h and there was no loading dose. Additionally, study patients received i.v. ketorolac (30 mg) every 6 h × 48 h.

A random number table was used by medical center pharmacy staff to assign study patients to receive either 0.5% bupivacaine + 5 mcg/ml epinephrine (1:200,000) or saline solution (both were clear liquids of equal volume), provided in identical-appearing pre-filled syringes. Content of the syringes used in this study could not be ascertained from labeling, and was registered only by numerical code secured in the pharmacy.

### Nerve block technique

Bilateral ilioinguinal and iliohypogastric nerve block (IINB) was placed by the surgeon in a two-stage fashion: the first component was administered 5 min before initial skin incision via 20-gauge needle (Stimuplex^® ^STIM-A150, B. Braun Medical Inc.; Bethlehem, Pennsylvania 18018 USA) with injection at the point 2.5 cm medial to the anterior superior iliac spine (ASIS) and 1 cm cephalad toward a reference line connecting umbilicus and ASIS [5]. The blunt portion of the needle permitted identification of fascia and served to push away peripheral nerves present in the loose connective tissue between muscle layers. The needle was advanced until a loss of resistance was perceived upon piercing external oblique fascia. After a negative aspiration test, an injection (4 ml) was carried out in a fanlike manner, interstitial to external and internal oblique muscle layers. This same technique was next used to deliver another 4 ml of solution between the internal oblique and transversus abdominis muscles.

The second component of the IINB was administered by injecting 8 ml of the same solution after fascial closure (using the same needle described above, at a 45° angle) to a point 2.5 cm medial to the ASIS. 4 ml of solution was injected between external and internal oblique muscle, and 4 ml of solution was placed between internal oblique and transversus abdominis mm., both in a fanlike pattern.

### Post-operative evaluation

Post-operative pain intensity was evaluated by a visual analogue score (VAS), where 0 = no pain to 10 = maximum/intolerable pain. Pain scores were registered at 2 h intervals by nursing staff until PCA was discontinued.

Morphine was given (up to 12 mg, as bolus) until patients were comfortable and VAS score was <3. Supplementary i.v. fentanyl was provided for refractory pain. Total cumulative dose of i.v. morphine sulfate from PCA was measured, and nausea, emesis and pruritus at 6, 24 and 48 h post-operatively were also recorded. Study patients' overall satisfaction with postsurgical pain management was reported as "1" if satisfied and as "2" if not satisfied.

### Statistical analysis

Two sided Student's *t*-test was used to compare mean data from the two groups, including those where dichotomous data were gathered [7]. Differences with *p *< 0.05 were considered significant.

## Results

A total of 61 patients were initially recruited, with 29 randomized to the bupivacaine group (Group I) and 32 to the saline (placebo) group (Group II). Patient age, body mass index, preoperative ASA (American Society of Anesthesiologists) class, and total operative duration were comparable between the two groups as shown in Table 2. One study patient in Group I was excluded because she was given a nonstandard, unapproved analgesic.

**Table 2:** Comparison of selected clinical features and perioperative characteristics among patients randomized to ilioinguinal-iliohypogastric nerve block (Group I) or saline control (Group II).

	Group I (*n *= 28)	Group II (*n *= 32)	*p*^1^
Age (yrs)	43.6 ± 8.4	39.9 ± 6.9	0.06
BMI^2^	29.6 ± 6.2	31.0 ± 5.8	0.39
ASA class^3^	1.8 ± 0.4	1.9 ± 0.5	0.37
Duration of surgery (min)	109.5 ± 44.2	106.2 ± 44.9	0.77
PCA^4 ^use (h)	27.4 ± 6.5	25.0 ± 4.2	0.09
Oral analgesic start time (h)	23.3 ± 3.6	22.8 ± 5.9	0.73
LOS^5 ^(h)	48.5 ± 13.2	49.4 ± 16.6	0.81

*Notes*: All data reported as mean ± SD; min = minutes, h = hours,

^1^by Student's *t*-test ^2^body mass index (kg/m^2^) ^3^American Society of Anesthesiologists class [as prognostic measure of perioperative morbidity] ^4^patient-controlled analgesia ^5^length of stay.

Table 2 shows mean time to initiate oral analgesics was 22.8 h for Group II vs. 23.3 h for Group I (*p *= 0.73), and average LOS for these two groups was 49.4 h hours and 48.5 h, respectively (*p *= 0.81). VAS for post-operative pain was similar between the two groups when pain intensity score was assessed by nurses (Table 3). The average quantity of morphine SO_4 _used in PACU was also similar among study patients as depicted in Table 4, irrespective of IINB (7.8 mg in Group I vs. 8.4 mg in Group II; *p *= 0.52). Additionally, PCA utilization and total morphine SO_4 _consumption was similar (47.3 in Group I vs. 45.9 mg in Group II; *p *= 0.85). When PCA use was stratified by post-surgical interval, the two study groups showed a consistent pattern of morphine SO_4 _consumption. Specifically, comparisons of PCA use in the first 8 h after surgery, the interval 8–16 h after surgery, and the interval 16–24 h after surgery revealed no significant differences between groups (*p *= 0.88, 0.93, and 0.53 respectively). Mean time until PCA discontinuation was also similar between the two groups (27.3 h in Group I vs. 24.9 h in Group II; *p *= 0.09). In PACU, three patients in the placebo arm (Group II) requested fentanyl in addition to morphine for pain control, while none in Group II required supplementation (data not shown). No significant differences were reported in itching, nausea, or vomiting between the two groups and both groups indicated an equivalent level of satisfaction with post-operative pain management (Table 5).

**Table 3:** Mean scores depicting post-operative pain intensity as measured by a visual analogue score recorded by nurses from patients randomized to ilioinguinal-iliohypogastric nerve block (Group I) or saline control (Group II).

*t *(h)	Group I (*n *= 28)	Group II (*n *= 32)	*p*^1^
2	4.67	5.17	0.51
4	3.64	3.60	0.95
6	3.66	3.01	0.38
8	2.62	2.71	0.89
10	2.36	2.70	0.55
12	2.24	2.26	0.96
14	1.94	1.93	0.98
16	1.48	2.06	0.26
18	1.82	1.69	0.80
20	1.63	1.69	0.90
22	1.63	2.36	0.26
24	1.86	2.25	0.53

*Notes*: *t *(h) = hours after surgery ^1^by Student's *t*-test.

**Table 4:** Summary of post-operative morphine use (bolus and PCA dosing) among patients randomized to ilioinguinal-iliohypogastric nerve block (Group I) or saline control (Group II).

	Group I (*n *= 28)	Group II (*n *= 32)	*p*^1^
PACU MSO_4 _bolus	7.8 ± 3.7	8.4 ± 3.7	0.52
MSO_4 _via PCA (total)	47.3 ± 25.8	45.9 ± 34	0.85
MSO_4 _via PCA (first 24 h)	41.7 ± 19.6	42.5 ± 34.8	0.91
initial 8 h	20.9 ± 10.5	20.4 ± 13.9	0.88
8–16 h	10.7 ± 7.4	11.0 ± 14.3	0.93
16–24 h	10.1 ± 7.1	11.7 ± 21.2	0.53
MSO_4 _via PCA (>24 h)	5.8 ± 9.1	2.2 ± 4.8	0.06

*Notes*: All data reported as mean ± SD (mg); PACU = post-anesthesia recovery unit, MSO_4 _= morphine sulfate ^1^by Student's *t*-test.

**Table 5:** Comparison of overall pain control effectiveness and selected analgesia-associated symptoms measured preoperatively and at various intervals after surgery among patients randomized to ilioinguinal-iliohypogastric nerve block (Group I) or saline control (Group II).

		Group I (*n *= 28)	Group II (*n *= 32)	*p*^1^
Pruritus	*t *= 0	1.00	1.06	0.35
	PACU	1.00	1.00	1.00
	6 h	1.17	1.12	0.56
	24 h	1.25	1.15	0.37
	48 h	1.03	1.03	0.92
				
Nausea/emesis	*t *= 0	1.00	1.03	0.35
	PACU	1.03	1.18	0.06
	6 h	1.25	1.25	1.00
	24 h	1.17	1.34	0.15
	48 h	1.03	1.12	0.21
				
Overall satisfaction	*t *= 0	1.03	1.09	0.37
	PACU	1.57	1.46	0.43
	6 h	1.10	1.12	0.83
	24 h	1.03	1.06	0.64
	48 h	1.03	1.00	0.28

*Notes*: All data tabulated as mean (1 = not present; 2 = present [for pruritus and nausea/emesis], 1 = satisfied; 2 = not satisfied [for overall satisfaction]); *t *= 0 is 'preoperative', PACU = post-anesthesia recovery unit, h = hours after surgery ^1^by Student's *t*-test

## Discussion

Pain after surgery has both somatic and visceral components and can be effectively relieved with neuraxial or systemic narcotics [4]. Somatic (cutaneous) pain generated from a Pfannensteil incision is principally conducted by the iliohypogastric and ilioinguinal nerves supplying afferent coverage to the L1–2 dermatome [8]. Suboptimal analgesia accounts for considerable patient dissatisfaction, prolonged LOS, and delayed return to normal daily activity. Post-operative wound pain may be reduced by infiltration of local anesthetic into the wound before closure [9-11]. Others have found preemptive local anesthetic nerve block to be useful in reducing post operative pain in both minimally invasive surgery and "open" laparotomy cases [12-17]. Our study enrolled women undergoing laparotomy for selected gynecologic indications and prospectively evaluated the efficacy of a dual-stage IINB comprising a preemptive and pre-closure component in this population.

A related study [18] involving hysterectomy patients observed a >50% decrease in morphine consumption in the initial 48 h after surgery when simple ilioinguinal block was performed. In that population, no significant difference in pain scores was seen when nerve block patients were compared to controls, a finding in agreement with our VAS data reported here.

Because decreased postoperative pain has been reported to result from infiltration given preoperatively or from infiltration nerve block before the end of the procedure [19-22], we hypothesized that a combination of both methods including a preemptive and an intraoperative preclosure infiltration would yield superior postoperative pain control. Indeed, our study tested a 30 ml (total volume) bupivacaine + epinephrine solution for more prolonged effect. Our investigation, however, did not identify a statistically significant difference in PCA morphine pump use among patients receiving saline controls or IINB. This finding was comparable to data reported among Cesarean delivery [23] and herniorrhaphy patients [24], where postoperative morphine use was not modified by administration of a one-stage, single-site injection.

A possible explanation for these observations may be found in the details of the surgeries studied. For example, the different post-operative analgesia requirements after Cesarean delivery [25] may be related to different pain modalities associated with that surgery, where somatic nociception predominates (*i.e*., less viscero-peritoneal stimulus). Thus, efficacy of preemptive anesthesia may depend on the type of procedure performed as suggested by Aïda *et al *[26], where it had little impact when done before gastrectomy, appendectomy or hysterectomy.

Although this is the first randomized placebo-controlled evaluation of the effect of combined preemptive and preclosure IINB in gynecologic surgery through a Pfannenstiel skin incision, it has some important limitations which must be noted. While our study was not powered to determine the minimum number of patients required to minimize Type II error, our sample size was influenced by an earlier investigation of 40 hysterectomy patients which was sufficient to detect a significant difference in postoperative morphine use as well as pain measured by VAS [4]. Data from the present research was not able to reproduce this finding, however, despite the increased sampling in our study. Additionally, IINB was not performed by the same surgeon thereby introducing some operator variability. Further prospective studies incorporating larger patient numbers are planned at our institutions to refine the role of IINB in pain control following gynecologic surgery.

In conclusion, data from this population do not support a clinically important role for two-stage IINB after some inpatient gynecologic procedures. Additional studies with larger sampling to better characterize post-operative pain management are planned at our institutions.

## Competing interests

The authors declare that they have no competing interests.

## Authors' contributions

SAW, LMG and EHD collected patient data and performed the surgeries; SLH and SLS supervised the research; CME designed the study and provided statistical analysis; ESS coordinated the study and drafted the manuscripts.
